# Genomic data of a multidrug-resistant *Klebsiella quasipneumoniae* strain NGET6B isolated from tannery wastewater in Bangladesh

**DOI:** 10.1016/j.dib.2026.113371

**Published:** 2026-10-30

**Authors:** Md. Mashiur Rahman, Fahad Khan, Abdus Sadique, Jahidul Alam, Katha Saha, Nudrat Nakib, Syed Raian Abedin, Md Azmain Faik, Shoheli Asrafie, S.M. Shihab, Ananya Saha, Fariza Shams, Ayesha Sharmin, Mohammad Moshiur Rahman, Md. Mahbubur Rahman, Md. Mainul Hossain, Maqsud Hossain

**Affiliations:** a NSU Genome Research Institute (NGRI), North South University, Dhaka, 1229, Bangladesh; b Department of Microbiology, North South University, Dhaka, 1229, Bangladesh; c Department of Biochemistry and Biotechnology, North South University, Dhaka, 1229, Bangladesh; d Department of Chemistry, Bangladesh University of Engineering & Technology, Dhaka, 1000, Bangladesh; e Department of Environmental Science and Management, Dhaka, 1229, Bangladesh; f Department of Pharmaceutical Sciences, North South University, Dhaka, 1229, Bangladesh

**Keywords:** *Klebsiella quasipneumoniae*, Antimicrobial resistance, Whole-genome sequencing

## Abstract

*Klebsiella quasipneumoniae* is increasingly recognized as a clinical pathogen and an environmental reservoir for antimicrobial resistance. Here we report the draft genome sequence of *K. quasipneumoniae* strain NGET6B, isolated from tannery wastewater in Narayanganj, Bangladesh. The assembled draft genome is 5812,357 bp in length with an N50 of 1.67 Mb and a GC content of 57.11%. Genomic analysis identified the species-intrinsic β-lactamase gene *bla*OKP-A-9, the mobilized colistin resistance gene *mcr-9*, and multiple virulence determinants including adhesion (type 1 *fim* and type 3 *mrk* fimbriae), capsule biosynthesis (*galF, gndA, ugd, rcsB*), iron acquisition through the enterobactin siderophore system (*ent/fep* genes), and a complete type VI secretion system. This genomic dataset provides valuable insights into the convergence of multidrug resistance and virulence in environmental isolates and highlights the role of industrial wastewater as a potential reservoir for clinically relevant pathogens.

Specifications Table

**Table:** 

Feature	Value
Subject	Microbiology
Specific subject area	Genomics, Molecular Microbiology, Environmental Microbiology
Type of data	Whole-genome sequence data (FASTA format), analyzed text, figures.
Data collection	Tannery effluent was collected in a sterile sampling bottle and transported to the laboratory in a cooler box. An aliquot was enriched in Luria-Bertani (LB) broth at a 1:10 (v/v) sample-to-broth ratio in a 15 mL Falcon tube and incubated overnight at 37 °C. The enriched culture was then streaked onto MacConkey agar and incubated overnight at 37 °C. A single, distinct pink, mucoid colony, presumptively identified as Klebsiella based on colony morphology, was subcultured on MacConkey agar to obtain a pure isolate. Genomic DNA was extracted from a single suspected Klebsiella colony grown overnight in LB broth media using the Promega Wizard® Genomic DNA Purification Kit. Sequencing was performed on the Illumina MiSeq platform (2 × 150 bp) with DNA library prepared with Nextera XT DNA Library Preparation Kit (Illumina, USA). Raw reads were quality checked with FastQC and trimmed with Trimmomatic v0.39 and assembled using SPAdes v3.15.0. Annotation was conducted via the NCBI Prokaryotic Genome Annotation Pipeline (PGAP) v6.11.
Data source location	*Klebsiella quasipneumoniae* strain NGET6B was isolated from a tannery effluent discharge point in Narayanganj, Dhaka, Bangladesh (23°37′21.1″N, 90°28′54.9″E)
Data accessibility	Direct link to Whole Genome Data: https://www.ncbi.nlm.nih.gov/nuccore/JBXEOM000000000.1/ Direct link to BioProject: https://www.ncbi.nlm.nih.gov/bioproject/PRJNA1285623/ Direct link to BioSample: https://www.ncbi.nlm.nih.gov/biosample/SAMN49770378/ Direct link to Raw Sequencing Data: https://www.ncbi.nlm.nih.gov/sra/SRR35147774

## 1 Value of the Data

• This dataset provides genomic insights into a multidrug-resistant environmental isolate of *K. quasipneumoniae*, addressing a scarcity of genomic data from industrial wastewater sources in Bangladesh.
• The genomic data will be highly beneficial for researchers tracking the horizontal gene transfer of significant antimicrobial resistance genes, such as the *mcr-9* mobilized colistin resistance gene and IncFIB plasmid replicons, in environmental settings.
• The identification of a diverse virulome, including the salmochelin iron acquisition system and type 1/3 fimbriae, offers a genetic basis for evaluating the persistence, environmental adaptability, and pathogenic potential of environmental *Klebsiella* strains.

## 2 Background

*Klebsiella quasipneumoniae* belongs to the *Klebsiella pneumoniae* species complex (KpSC) and has emerged as both an opportunistic pathogen and a reservoir for antimicrobial resistance (AMR) [1,2]. While generally considered less virulent than *K. pneumoniae*, some strains produce extended-spectrum beta-lactamases (ESBLs) and hypermucoviscous traits, resulting in comparable infection rates [1,3]. *K. quasipneumoniae* strains have increasingly been detected in environmental sources, raising public health concerns about AMR transmission through direct contact or the food chain [4]. Despite their potential clinical significance, there is a scarcity of data on *Klebsiella quasipneumoniae* from environmental sources, particularly industrial wastewater. Tannery effluent is of particular concern, as it combines heavy metals, high organic load and residual chemicals that can co-select for antimicrobial resistance and favor the exchange of mobile genetic elements among bacteria [5]. Here, we report the draft genome of a *Klebsiella quasipneumoniae* strain NGET6B, isolated from a tannery effluent, with a focus on its antimicrobial resistance determinants, virulence-associated genes, and overall genomic features. The dataset provides insights into the resistance and virulence potential of an environmental *K. quasipneumoniae* isolate and contributes to the genomic surveillance of environmental antimicrobial resistance within a One Health framework.

## 3 Data Description

Genome sequencing of the strain produced a total of 1183,486 paired raw reads which were quality-checked and trimmed prior to genome assembly. The assembled draft genome of *K. quasipneumoniae* strain NGET6B spans 5812,357 base pairs and comprises 1055 contigs assembled into 303 scaffolds, with an N50 of 1.67 Mb, an L50 of 2, and a GC content of 57.11% (Table 1). FastANI analysis confirmed its taxonomic assignment, revealing an OrthoANIu value of 98.51% nucleotide identity to the *K. quasipneumoniae* reference genome GCF_020099175.1 (Fig. 1). Phylogenomic analysis in Type (Strain) Genome Server (TYGS) through the Genome BLAST Distance Phylogeny (GBDP) approach confirmed strain NGET6B as *K. quasipneumoniae*, clustering with reference strain 01A030 (node support = 100) and distinctly separating from related *K. pneumoniae* and *K. variicola* clades (Fig. 2). The phylogenomic tree was visualized using the newick file from TYGS in Interactive Tree of Life (iTOL) [6]. Multilocus sequence typing (MLST) was performed using BIGSdb v1.52.2 [7] which assigned the strain to the nearest sequence type, ST 9253 (loci matched 6/7 (85.7%) (Table 2)

**Table 1:** Genomic characteristics of *Klebsiella quasipneumoniae* strain NGET6B.

Features	Value
Genome Length	5812,357 bp
GC %	57.1%
Genome Coverage	∼30x
Scaffolds	303
Longest Scaffold Size	2162596
N50	1671688
N90	14601
L50	2
L90	10
Genes (Total)	6493
CDSs (Total)	6441
CDSs (with protein)	5460
Genes (RNA)	52
tRNA	39
rRNA	2, 1, 1 (5S, 16S, 23S)
ncRNA	9

**Fig. 1:**
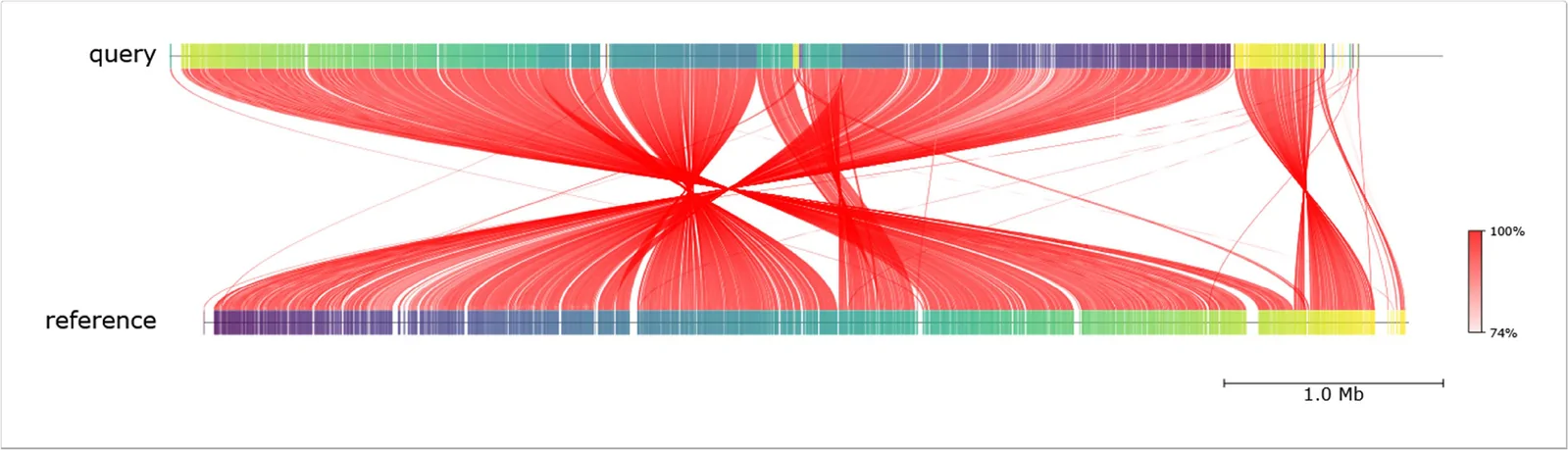
Average Nucleotide identity (ANI) showing the similarity between *Klebsiella quasipneumoniae* strain NGET6B (query) and reference genome.

**Fig. 2:**
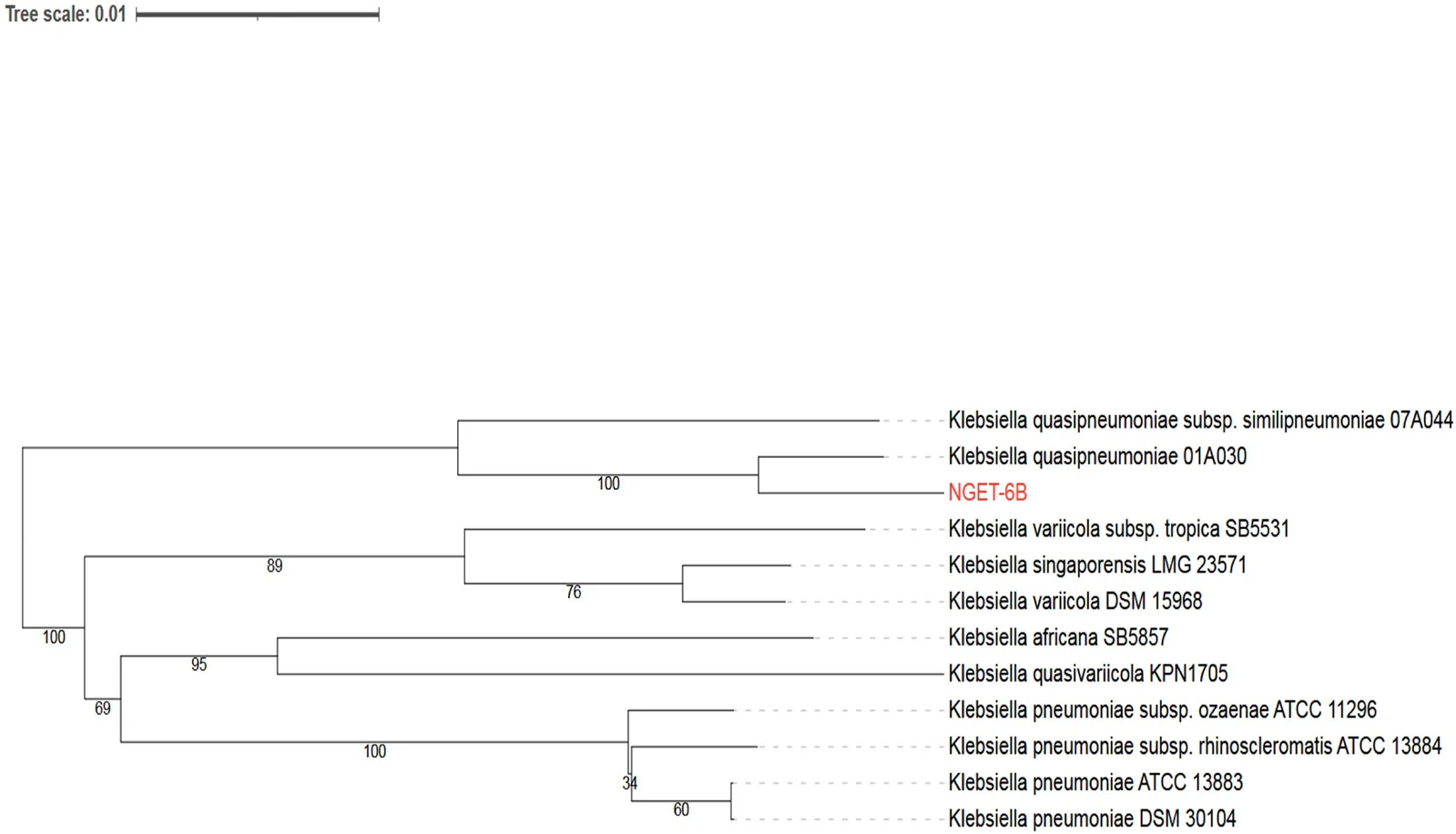
Whole-genome-based phylogenetic tree showing the evolutionary relationships of *Klebsiella quasipneumoniae* strain NGET6B with closely related Klebsiella species. Branch lengths are scaled according to the GBDP distance formula d5, with a scale bar representing an evolutionary distance of 0.01. Numbers at internal nodes indicate GBDP pseudo-bootstrap support percentages calculated from 100 replicates.

**Table 2:** MLST profile of *Klebsiella quasipneumoniae* strain NGET6B assigned to Nearest sequence type: ST-9253 (BIGSdb Version 1.52.2).

Locus	Allele	Length	Contig	Start position	End position
*gapA*	17	450	Scaffold_2	150794	151243
*infB*	55	318	Scaffold_8	516247	516564
*mdh*	39	477	Scaffold_8	450683	451159
*pgi*	603	432	Scaffold_11	103065	103496
*phoE*	117	420	Scaffold_2	1204320	1204739
*rpoB*	18	501	Scaffold_11	157428	157928
*tonB*⁎	-	-	-	-	-

⁎ The *tonB* locus was uncalled in the draft assembly of strain NGET6B.

NCBI PGAP v6.11 annotation identified a total of 6493 genes, comprising 5460 protein-coding sequences and 52 RNAs (4 rRNAs and 39 tRNAs) (Table 1). RAST-based subsystem annotation [8] assigned genome features of strain NGET6B to 383 distinct subsystems, accounting for 28% of annotated coding sequences (Fig. 3). Among the classified features, genes involved in carbohydrate metabolism (492 features) and amino acid and derivative metabolism (466 features) were the most highly represented categories, followed by protein metabolism (227), cofactors, vitamins, prosthetic groups and pigments (209), and respiration (163). Categories of particular relevance to environmental persistence and pathogenic potential were also well represented, including stress response (104 features), virulence, disease and defense (69 features), and iron acquisition and metabolism (46 features).

**Fig. 3:**
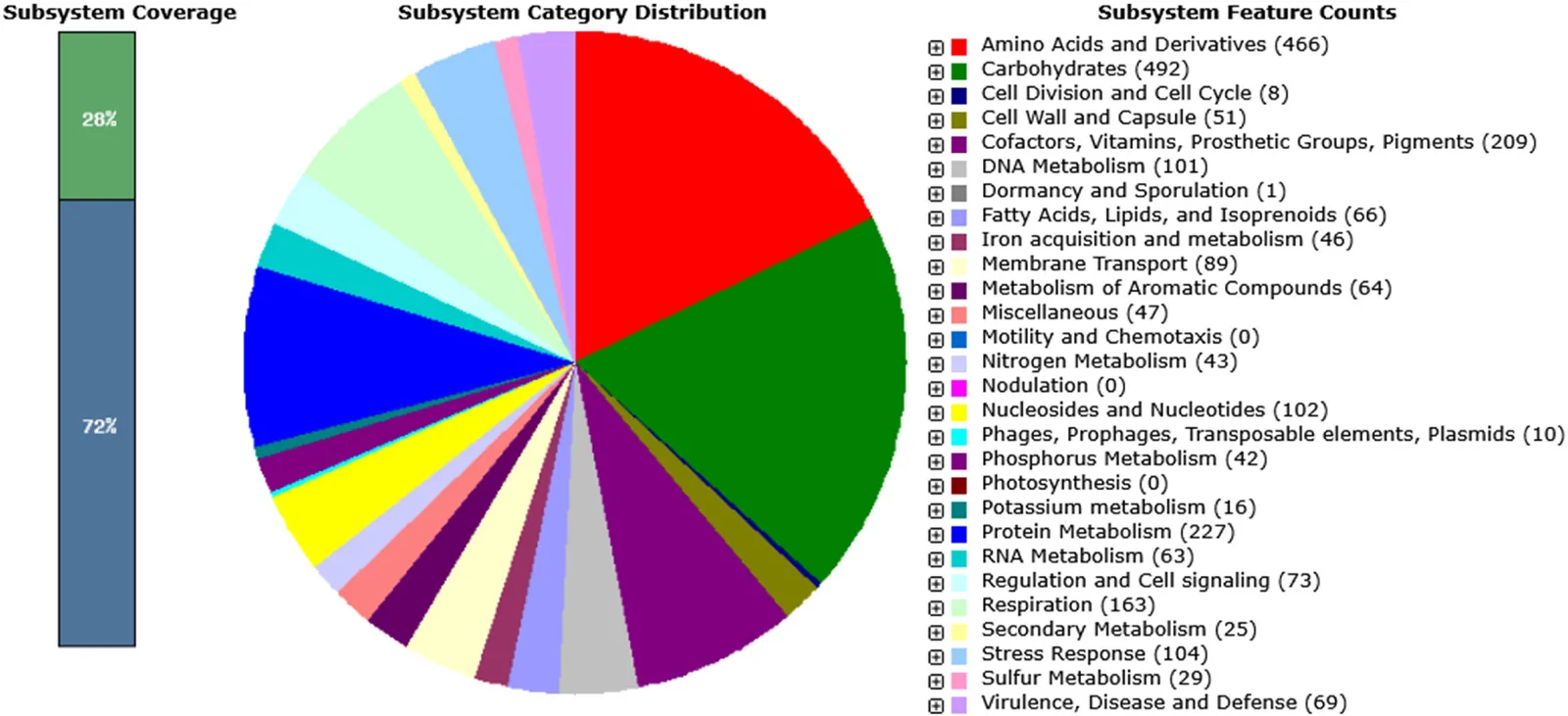
Functional subsystem annotation of *Klebsiella quasipneumoniae* strain NGET6B.

ResFinder v4.7.2 revealed a diverse array of antimicrobial resistance determinants (Table 3). The isolate harbors the species-intrinsic beta-lactamase gene *blaOKP-A-9* alongside the mobilized colistin resistance gene *mcr-9*. Additionally, ResFinder identified the fosfomycin resistance genes *fosA* and *fosA6*, as well as the multidrug efflux pump components *oqxA* and *oqxB*, which mediate resistance to quinolones, folate pathway antagonists, and amphenicols.

**Table 3:** Antimicrobial Susceptibility genes identified in *Klebsiella quasipneumoniae* strain NGET6B by ResFinder.

Resistance Gene	Drug Class	Resistance Mechanism	Antimicrobials	Identity
blaOKP-A-9	beta-lactam	Antibiotic inactivation	Penicillins	100.00%
mcr-9	polymyxin	Antibiotic target alteration	Colistin	99.28%
fosA6	fosfomycin	Antibiotic inactivation	Fosfomycin	92.14%
fosA	fosfomycin	Antibiotic inactivation	Fosfomycin	92.14%
OqxB	quinolone, folate pathway antagonist, amphenicol	Antibiotic efflux	Ciprofloxacin, Nalidixic acid, Trimethoprim, Chloramphenicol	94.99%
OqxA	quinolone, folate pathway antagonist, amphenicol	Antibiotic efflux	Ciprofloxacin, Nalidixic acid, Trimethoprim, Chloramphenicol	94.47%

PlasmidFinder v3.0.3 identified IncFIB(K), IncFIB(pNDM-Mar), and RepB replicons (Table 4). The presence of IncFIB plasmids is highly notable as they are often reported alongside NDM-encoding genes in Enterobacterales. In one study in Bangladesh, a conjugative IncFIB(pQil) plasmid carried mcr-8.1, blaCTX-M-15 and other resistance genes in *K. pneumoniae* (ST15) obtained from hospital infections [9]. In another study in Thailand, all 15 ST16 isolates from a hospital collection of blaNDM-carrying, carbapenem-resistant *K. pneumoniae* (CRKP) carried IncFIB(pQil) alongside blaNDM-1 and blaOXA-232 [10]. The co-occurrence of IncFIB replicons, mcr-9 and virulence-associated genes in NGET6B resembles clinical Klebsiella profiles and is consistent with a One Health link between tannery effluent and hospital settings. However, the short-read draft assembly in this study cannot show whether mcr-9 is plasmid-borne with these replicons, which would require long-read sequencing.

**Table 4:** Identified plasmids in the strain.

Plasmid factor	Identity	Query / Template length	Contig	Position in contig	Accession no.
IncFIB(K)	98.93%	560 / 560	Scaffold_14	6350..6909	JN233704
RepB	99.32%	438 / 438	Scaffold_183	5773..6210	CP061702.1
IncFIB(pNDM-Mar)	99.77%	439 / 439	Scaffold_280	1096..1534	JN420336

VFDB analysis identified **33 virulence-associated genes** in the strain (Supplementary File 01), representing a diverse set of functional categories associated with bacterial adhesion, biofilm formation, capsule-associated functions, iron acquisition, efflux, and secretion. Genes encoding type I fimbriae, including *fimA, fimD, fimG, fimH,* and *fimI*, as well as type III fimbrial components (*mrkA–F* and *mrkH*), were detected, indicating the presence of genetic determinants associated with surface attachment and biofilm formation. Several genes involved in capsule biosynthesis and regulation, including *galF, KP1_RS17355, gndA, ugd,* and *rcsB*, were also identified. The strain possessed an extensive repertoire of enterobactin-associated iron acquisition genes, including *ent, fep,* and *fes* loci. In addition, *acrA*, a component of the AcrAB efflux system, and several genes associated with the type VI secretion system were detected.

## 4 Experimental Design, Materials and Methods

Tannery wastewater sample was collected from a tannery effluent discharge point in Narayanganj, Dhaka, Bangladesh (23°37′21.1″N, 90°28′54.9″E) during March 2018. The sample was enriched in LB broth at 37 °C for 18–20 h and then cultured on MacConkey agar (Oxoid, UK) media and incubated overnight at 37 °C. A single lactose-fermenting, mucoid colony designated NGET6B was selected for further analysis.

A single distinguished colony from a fresh culture was used for DNA extraction after incubation in Luria-Bertani (LB) broth overnight at 37 °C. Genomic DNA was extracted using the Promega Wizard® Genomic DNA Purification Kit following the manufacturer’s instructions. The sequencing library was prepared with the Nextera XT DNA Library Preparation Kit (Illumina Inc, USA). Whole-genome sequencing was conducted on an Illumina MiSeq platform yielding 2 × 150 bp reads using the MiSeq Reagent Kit v2 (300 cycles) (Illumina Inc, USA).

Sequence reads were quality-checked and trimmed using FastQC v12.1 [11] and Trimmomatic v0.39 (Q ≥ 30) [12] and assembled de novo with SPAdes v3.15.0 [13]. Genome annotation was executed using the NCBI PGAP v6.11 [14]. Species identity was verified using KmerFinder v3.2, and genomic similarity was assessed via FastANI analysis [15] through the EZBioCloud platform (https://www.ezbiocloud.net/tools/ani). Taxonomic identification of strain NGET6B was performed using the Type (Strain) Genome Server (https://tygs.dsmz.de) [16] via Genome BLAST Distance Phylogeny (GBDP) under default settings. Phylogenomic trees were built using FastME with 100 bootstrap replicates, applying the 70% dDDH cutoff for species delimitation. Plasmids were identified utilizing PlasmidFinder v3.0.3 [17]. Resistance and virulence determinants were predicted using ResFinder v4.7.2 [18] (≥90% identity, ≥60% length) and Virulence Factor Database (VFDB; 2024 update) [19] using BLASTn with a minimum identity threshold of 90%.

## Limitations

Not applicable.

## Credit Author Statement

**Md. Mashiur Rahman:** Formal Analysis, Data curation, Visualization, Writing – review & editing. **Fahad Khan:** Methodology, Software, Writing – original draft, Writing – review & editing. **Jahidul Alam:** Resources, Formal Analysis, Methodology. **Abdus Sadique:** Resources, Methodology. **Katha Saha:** Methodology, Writing – review & editing. **Nudrat Nakib:** Methodology, Software. **Syed Raian Abedin:** Methodology, Formal Analysis. **Md Azmain Faik:** Formal Analysis, Writing – review & editing. **Shoheli Asrafie:** Formal Analysis, Methodology. **S M Shihab:** Formal Analysis, Methodology. **Ananya Saha:** Formal Analysis, Methodology. **Fariza Shams:** Formal Analysis, Validation, Supervision. **Ayesha Sharmin:** Formal Analysis, Validation, Supervision. **Mohammad Moshiur Rahman:** Supervision, Formal Analysis. **Md. Mahbubur Rahman:** Formal Analysis, Supervision. **Md. Mainul Hossain:** Conceptualization, Methodology, Fund acquisition, Supervision. **Maqsud Hossain:** Conceptualization, Methodology, Validation, Supervision, Writing – review & editing.

## Ethics Statement

The bacterial isolate analyzed in this study was obtained from tannery effluent in Narayanganj and did not involve human participants, clinical specimens, or identifiable personal data. Therefore, ethical approval and informed consent were not required for this study.

## Data Availability

All data generated in this study are fully released and publicly accessible through NCBI under the accession numbers mentioned below. The whole-genome sequence is deposited in NCBI GenBank under accession JBXEOM000000000. The sequence reads are available in the NCBI SRA under accession SRR35147774, linked to BioProject PRJNA1285623 and BioSample SAMN49770378.

## Appendix Supplementary materials

Image, application 1
